# Predictive Value of the Hemoglobin-Geriatric Nutritional Risk Index in Patients with Heart Failure

**DOI:** 10.3390/nu15224789

**Published:** 2023-11-15

**Authors:** Momoko Tohyama, Yuka Shirai, Miho Shimizu, Yuki Kato, Yoji Kokura, Ryo Momosaki

**Affiliations:** 1 Department of Rehabilitation Medicine, Mie University Graduate School of Medicine, Tsu 514-8507, Japan; 323m005@o.mie-u.ac.jp (M.T.); yunnkaa@gmail.com (Y.S.); yukikato698@gmail.com (Y.K.); 2 Department of Nutrition, Hamamatsu Medicine University Hospital, Hamamatsu 431-3192, Japan; 3 Department of Rehabilitation, Mie University Hospital, Tsu 514-8507, Japan; m-shimizu@med.mie-u.ac.jp; 4 Department of Nutritional Management, Keiju Hatogaoka Integrated Facility for Medical and Long-Term Care, Hosu 927-0023, Japan; yojikokura@hotmail.com

**Keywords:** malnutrition, heart failure, Hemoglobin-Geriatric Nutritional Risk Index, Geriatric Nutritional Risk Index, Barthel Index, prognosis, hospitalization-associated disability

## Abstract

Malnutrition prevails among patients with heart failure (HF), increasing the likelihood of functional decline. We assessed the predictive value of the Hemoglobin-Geriatric Nutritional Risk Index (H-GNRI)—combining hemoglobin and the Geriatric Nutritional Risk Index (GNRI)—on prognosis in older patients with HF. We used the JMDC multicenter database to examine the potential associations between malnutrition risk and other outcome measures. The patients were categorized as low- (H-GNRI score = 2), intermediate- (H-GNRI score = 1), or high-risk (H-GNRI score = 0) based on their H-GNRI scores. The primary outcome measure was the Barthel Index (BI) gain; the secondary outcomes included the BI at discharge, the BI efficiency, length of hospital stay, in-hospital mortality, discharge to home or a nursing home, and hospitalization-associated disability. We analyzed 3532 patients, with 244 being low-risk, 952 being intermediate-risk, and 2336 being high-risk patients. The high-risk group of patients had significantly lower BI values at discharge, lower BI gains, reduced BI efficiency values, and prolonged hospital stays compared to those in the low-risk group. The high-risk patients also had higher in-hospital mortality rates, lower rates of discharge to home or a nursing home, and greater incidences of a hospitalization-associated disability in comparison to the low-risk group. The H-GNRI may serve as a valuable tool for determining prognoses for patients with HF.

## 1. Introduction

Heart failure (HF) is a clinical condition characterized by reduced cardiac pump function resulting from cardiac dysfunction, leading to decreased exercise tolerance. The global prevalence of HF exceeds 64 million patients [1], and its incidence rises with advancing age. Consequently, the patient population with HF is expected to continue growing as the world’s population ages [2]. Moreover, the total HF health care costs in the United States are expected to increase from USD 20.9 billion in 2012 to USD 53.1 billion in 2030, with approximately 80% of the total health care costs being related to hospitalizations [3]. The escalating medical expenses associated with prolonged hospitalization and recurrent readmissions in HF pose a serious challenge. Older patients with HF often experience a decline in Activities of Daily Living (ADL) following hospitalization, which has been linked to adverse outcomes, including mortality [4,5]. In addition, the decline in ADL associated with hospitalization is a serious problem that can increase the costs of care after discharge. An early assessment of the risk of ADL decline and early intervention are needed to prevent these problems.

Malnutrition is a common issue among patients with HF and it has been associated with a lower quality of life and unfavorable prognoses [6,7]. For instance, the Geriatric Nutritional Risk Index (GNRI), a tool used to assess nutritional risk status, has been evaluated for prognostic accuracy in patients with HF. Calculated based on the ratio of ideal weight and serum albumin levels, the GNRI has shown promise in predicting mortality and hospital stay duration in patients with HF [7,8,9]. However, some studies have reported that the GNRI does not predict in-hospital mortality or a decline in the Barthel Index (BI) among patients with HF [9,10]. Consequently, the GNRI’s prognostic value in patients with HF appears to be limited. The prognostic accuracy of the GNRI for post-hospitalization HF-related mortality has been estimated at approximately 0.70 in terms of the area under the curve [11], suggesting that there is room for improved diagnostic precision.

Recently, a new nutritional risk assessment index, the Hemoglobin-GNRI (H-GNRI), emerged [12]. The H-GNRI combines the GNRI with hemoglobin levels. A study published in 2021 by Wang et al. reported that the H-GNRI was an independent predictor of survival in patients with esophageal squamous cell carcinoma who underwent an esophagectomy and postoperative radiation therapy. The H-GNRI may be useful as a prognostic factor for diseases prone to malnutrition and low hemoglobin levels.

Anemia is common among patients with HF. The prevalence of anemia in patients hospitalized for HF has been reported to be about 50% [13]. Anemia in HF patients is multifactorial, and the primary causes include chronic inflammation, iron deficiency, and renal dysfunction related to cardiac problems [14,15]. In studies of patients with HF, lower hemoglobin levels have been reported to be associated with disease severity, re-hospitalization, and mortality in patients with HF [9,16,17]. Based on these findings, the H-GNRI, a composite index incorporating hemoglobin and the GNRI, may be valuable in predicting the prognosis for patients with HF who are prone to malnutrition and hemoglobin decline. We hypothesized that a nutritional risk assessment using the H-GNRI would effectively predict the prognosis in patients with HF. The objective of this study was to investigate the utility of the H-GNRI in forecasting outcomes among older patients with HF using a multicenter database.

## 2. Materials and Methods

### 2.1. Study Design and Ethics

This study is a historical cohort focusing on older patients with HF, sourced from the JMDC multicenter database. The ethics committee of Mie University deemed an ethical review unnecessary, citing the database’s established academic value and widespread use in research. In addition, informed consent was not required because all data obtained from the database were deidentified.

### 2.2. Data Source

The JMDC database contains medical reimbursement records, laboratory values, and Diagnostic Procedure Combination (DPC) data collected from multiple hospitals in all of Japan [18]. Introduced in Japan in 2003, the DPC is a medical payment system [19]. The DPC dataset includes a wide range of clinical information, admission and discharge statuses, diagnoses, surgeries and procedures, medications, and specialized reimbursement details pertaining to specific diseases [20].

We collected data on patients admitted for HF from January 2017 to June 2022 using the JMDC database. The database provides information on age, sex, body mass index (BMI) at admission, BI at admission and discharge, New York Heart Association (NYHA) classification, blood test values at the earliest within the first 5 days after admission, pre-admission residency information, use of ambulance services, post-discharge residency information, length of hospital stay, in-hospital mortality, the Charlson Comorbidity Index (CCI) score at admission, ventilator and vasopressor use, number of beds, and year of admission.

The patients were categorized into three age groups: pre-old (65–74 years), old (75–89 years), and oldest-old (≥90 years), as defined by the Japanese Geriatrics Society [21]. BMI was calculated by dividing body weight (kg) by height (m), squared and classified into <18.5, 18.5–24.9, 25.0–29.9, and ≥30 [22]. The Barthel Index (BI) was used to assess the ADL in patients; the BI consists of a total of 10 items, including feeding, bathing, grooming, dressing, bowels, bladder, toilet use, transfers (from bed to a chair and back), mobility (on level surfaces), and stairs, with a higher BI score indicating greater independence in ADLs [23]. The NYHA classification served as an exercise tolerance measure for patients with HF. The CCI quantifies 19 comorbid conditions contributing to mortality by summing their respective scores [24]. Ventilator and vasopressor use were documented from admission to the following day. The number of beds indicates the number of hospital beds in the hospital where each patient was admitted.

### 2.3. Participants

We included patients aged 65 or older admitted with HF (ICD-10 code: I50) between January 2017 and June 2022. The study cohort was limited to those residing in their own homes or a nursing home prior to admission. Patients who underwent open-heart procedures (such as bypass surgery, valvuloplasty, and valve replacement surgery) during their hospital stay were excluded, as were those with missing data for albumin, hemoglobin, BMI, BI, or post-discharge residency data.

### 2.4. Geriatric Nutritional Risk Index (GNRI)

Due to its validity [25], we set the ideal body weight as BMI 22 and calculated each patient’s GNRI as follows:
GNRI =14.84 × Albumin (g/dL) + 41.7 × weight (kg)/ideal body weight (kg) =14.84 × albumin (g/dL) + 41.7 × weight (kg)/[(height)^2^(m^2^)/22] =14.84 × albumin (g/dL) + 41.7 × BMI/22.


To prevent underestimating the nutritional risk for overweight patients, the patients’ BMI/22 were adjusted to 1 in cases where a patient’s BMI/22 was >1 [25].

### 2.5. Hemoglobin-GNRI (H-GNRI)

The H-GNRI classification comprises three groups, categorized by Wang et al.’s varying levels of nutritional risk [12]:

1. Low-risk group (H-GNRI score 2): normal GNRI and normal hemoglobin;
2. Intermediate-risk group (H-GNRI score 1): low GNRI or low hemoglobin;
3. High-risk group (H-GNRI score 0): low GNRI and low hemoglobin.

The GNRI and hemoglobin cutoff values were based on Wang et al., classified as low GNRI (<96), normal GNRI (≥96), low hemoglobin (<13.6 g/dL), and normal hemoglobin (≥13.6 g/dL) [12]. Since the patients in this study were >65 years of age, and based on previous studies [26,27], we considered the hemoglobin cutoff values to be acceptable for both men and women. Normal GNRI and normal hemoglobin were scored as 1, and low GNRI and low hemoglobin were scored as 0. The sum of the GNRI and hemoglobin scores was the H-GNRI score.

### 2.6. Outcomes

The primary outcome was BI gain, defined as the difference between BI at discharge and BI at admission. Secondary outcomes included BI at discharge, BI efficiency, length of hospital stay, in-hospital mortality, and discharge destination (home or nursing home). BI efficiency was calculated by dividing BI gain by the length of hospital stay. Hospitalization-associated disability was defined as a BI gain of less than zero.

### 2.7. Statistical Analysis

We are currently conducting a comparative analysis of the baseline data and outcomes among the low-risk, intermediate-risk, and high-risk groups. We present categorical data as absolute values and rates and employ χ^2^ tests to assess between-group differences. Continuous data are expressed as mean ± standard deviation, and we evaluate differences among the three groups through a one-way analysis of variance. To explore the associations between H-GNRI and BI at discharge, BI gain, BI efficiency, and length of hospital stay, we perform a multiple regression analysis. Additionally, a multiple logistic regression analysis is employed to investigate the relationships between H-GNRI and in-hospital mortality, discharge to home or nursing home, and hospitalization-associated disability. In addition, a subgroup analysis based on age was performed for the BI at discharge. In our multivariate analysis, we consider the following covariates: age, sex, NYHA class, BI at admission, CCI, ventilator use on admission, vasodilator use on admission, use of ambulance service, number of beds, and year of admission. All statistical analyses are carried out using SPSS software (version 19.0, IBM Japan, Tokyo, Japan), and statistical significance is defined as *p* < 0.05.

## 3. Results

**Figure 1:**
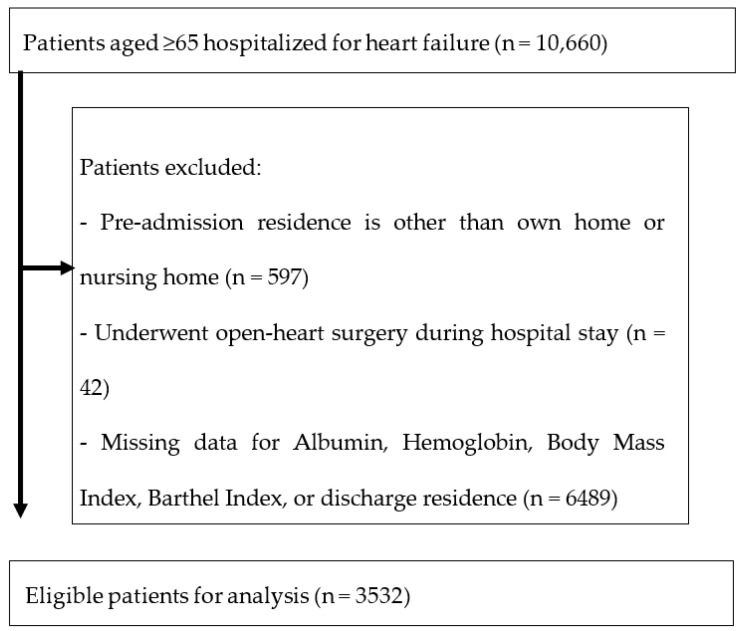
Patient selection process.

Among the 10,660 patients aged 65 or older who were hospitalized for HF, we excluded 7128 from the analysis for the various reasons detailed in Figure 1, leaving 3532 patients with HF for the analysis (Figure 1).

We divided the patients into low- (n = 244; 6.9%), intermediate- (n = 952; 27.0%), and high-risk (n = 2336; 66.1%) groups based on their respective H-GNRI scores. The patients’ background data are presented in Table 1. The high-risk group of patients tended to be older, female, and underweight, demonstrating a lower BI and higher CCI than the lower-risk patients.

Table 2 presents our between-group comparisons. The high-risk patients had lower BI values at discharge, BI gains, BI efficiency rates, and rates of discharge to home or a nursing home, longer hospital stays, higher in-hospital mortality rates, and higher incidences of a hospitalization-associated disability than the low- or intermediate-risk patients.

Table 3 shows the multiple regression analysis results. After adjusting for confounders, a high-risk status was independently associated with the BI at discharge (coefficient, −11.08; 95% Confidence Interval [CI], −15.29–−6.87), BI gain (coefficient, −11.08; 95% CI, −15.29–−6.87), BI efficiency (coefficient, −1.23; 95% CI, −1.87–−0.59), and length of hospital stay (coefficient, 5.29; 95% CI, 2.22–8.25).

Table 4 shows the multiple logistic regression analysis results. After adjusting for confounders, we found the high-risk group to be independently associated with in-hospital mortality (Odds Ratio [OR], 2.51; 95% CI, 1.28–4.90), discharge to home or a nursing home (OR, 0.42; 95% CI, 0.25–0.69), and hospitalization-associated disability (OR, 1.87; 95% CI, 1.18–2.97).

Table 5 shows the results of the subgroup analysis based on age for the BI at discharge. In the 65–74 age category, the high-risk group was not associated with BI at discharge (coefficient, −4.59; 95% CI, −11.59–2.41). The high-risk group was independently associated with BI at discharge in the 75–89 (coefficient, −14.11; 95% CI, −19.82–−8.14) and the > 90 years old categories (coefficient, −15.28; 95% CI, −27.46–−3.11).

## 4. Discussion

In this study, we assessed the utility of the H-GNRI in predicting the prognosis among older patients with HF using a multicenter database. We found that the H-GNRI score was associated with BI at discharge, BI gain, BI efficiency, length of hospital stay, in-hospital mortality, discharge to home or a nursing home, and hospitalization-associated disability. A high-risk H-GNRI was independently associated with these outcomes.

Table 6 shows a list of previous studies that examined the prognostic value of the GNRI in patients with HF. Table 6 shows a list of previous studies that examined the prognostic value of the GNRI in patients with HF. Half of the studies included HF patients with preserved ejection as the target population. Two studies (25%) defined the age of patients as 65 years or older, one study (12.5%) defined the age of patients as 80 years or older, and five studies (62.5%) did not define criteria based on age. The most common cutoff for the GNRI was 92, which was used in 75% of the studies. Patients on dialysis were excluded in half of the studies. Six studies (75%) set an outcome related to death, and two studies (25%) set an outcome related to ADLs. The GNRI is a very simple and objective nutritional risk assessment index that does not require specialized skills or user experience. These are the major advantages of the GNRI for clinical use. While prior studies examined its prognostic value and have demonstrated its independence in predicting mortality and prolonged hospital stay in patients with HF [7,8], there is concern about the impact of edema, which is prevalent in HF patients, on the GNRI [9]. We hypothesized that H-GNRI may predict prognosis in older HF patients.

The high-risk H-GNRI group exhibited a significantly lower BI gain than the other risk groups. After adjusting for confounders, the H-GNRI remained independently associated with BI gain. Furthermore, the mean BI gain (13.1) was greater than the minimal clinically important difference in BI for acute stroke (9.8 point) [33] and femoral neck fracture (9.25 point) [34], indicating a clinically meaningful change in the BI in this study. Regardless of the nutritional risk, the BI increased with general condition recovery from the inpatient treatment, although the high-risk H-GNRI group had a smaller BI gain than the other groups.

Previous research has shown a positive correlation between hemoglobin levels and ADL recovery in older hospitalized patients [35]. Anemia is also associated with reduced ADL in patients with HF [36,37]. Hemoglobin, a key component of red blood cells, is pivotal in oxygen transport. Reduced hemoglobin levels can lead to symptoms such as dizziness, palpitations, fatigue, and limited physical activity [38,39]. Patients in the high-risk H-GNRI group are more likely to have anemia, which may hinder ADL recovery due to the associated physical limitations.

Furthermore, GNRI—a component of the H-GNRI—is a nutritional risk assessment index calculated using BMI and albumin. Previous studies have highlighted that malnutrition poses a risk for hospitalization-associated disability in older hospitalized patients, with chronic-disease-related malnutrition linked to a lower BI at discharge [40,41]. A low BMI is also associated with post-discharge functional decline [42]. These findings suggest that malnutrition risk can impede rehabilitation effectiveness and negatively impact ADL recovery. In addition, malnutrition is a risk for complications such as infections [43]. Although this study could not be verified because it was not possible to detect all infective complications, higher-nutritional-risk patients may be at a higher risk for complications, which may have inhibited the recovery of ADLs.

The high-risk H-GNRI group had a higher incidence of hospitalization-associated disability than the other groups. After adjusting for confounders, hospitalization-associated disability was still independently associated with the H-GNRI score, suggesting that the H-GNRI may predict ADL decline during hospitalization. This could be explained for the same reason that the BI gains were smaller in the high-risk group, the effects of anemia, undernutrition, and comorbidities on ADLs.

However, the GNRI alone was insufficient to predict BI decline in the HF patients [10]. One possible explanation is that the nutritional risk assessment via the GNRI relies on body weight. Fluid retention and edema are common symptoms in patients with HF [44]. In cases in which patients have fluid retention, an assessment via body weight is not appropriate. Therefore, the GNRI may underestimate the nutritional risk of HF patients. Combining the GNRI with hemoglobin may address this underestimation, potentially enhancing the prognostic accuracy in older patients with HF.

The results of the subgroup analyses by age showed that high-risk H-GNRI was independently associated with BI at discharge in the 75–89 and >90 years old age categories. However, no association was found between the H-GNRI and BI at discharge in the 65–74 age group. This finding could be due to various reasons. First, the sample size decreased by dividing the sample into subgroups. Smaller sample sizes make it more difficult to detect statistically significant differences because the *p*-values are affected by the sample size. Second, the higher mean BI at discharge in the 65–74 age category may have caused a ceiling effect.

Assessments using a bioelectrical impedance analysis and dual energy X-ray absorptiometry have been reported to be associated with clinical outcomes in HF patients [45,46]. However, the use of a bioelectrical impedance analysis and dual-energy X-ray absorptiometry requires special equipment and much labor, and there is a high cost of installing the equipment. The H-GNRI is a simple nutritional risk assessment index suitable for clinical use that can be calculated using only the height, weight, and routinely measured blood test data of hospitalized patients without the need for special equipment, much expense, or labor.

This has limitations, including a lack of post-discharge information due to limits on retrospective data acquisition and potential selection bias, as the data were drawn from hospitals that submitted inpatient data. These hospitals may provide different care to patients compared to those that did not. Consequently, we cannot draw assumptions about the utility of the H-GNRI for long-term prognostication. In addition, we could not assess biomarkers such as natriuretic peptide, adipocytokines, and galectin 3, nor could we assess information on patient lifestyle habits such as alcohol and smoking. Additionally, we were unable to obtain data on indicators such as the bioelectrical impedance analysis, Subjective Global Assessment, and Nutritional Risk Screening 2002; thus, we were unable to make comparisons with the H-GNRI. Furthermore, this study demonstrated an association between the H-GNRI and clinical outcomes in patients with HF. However, it is not possible to determine these causal associations.

This study revealed the potential utility of the H-GNRI in predicting the prognosis of older patients with HF; however, long-term prognosis could not be validated. Thus, further research is still needed. A subsequent study is required to include extended follow-up periods to assess the long-term prognostic value of the H-GNRI in HF patients. It is possible to determine the most effective method to identify HF patients with or at risk of malnutrition by clarifying the utility of the H-GNRI and comparing it with other nutritional risk assessment indexes. In addition, interventions for high-risk patients based on nutritional risk assessment using the H-GNRI and their effectiveness need to be investigated.

## 5. Conclusions

Among older patients with HF, the H-GNRI scores were associated with the BI at discharge, BI gain, BI efficiency, length of hospital stay, in-hospital mortality, discharge to home or a nursing home, and hospitalization-associated disability. Thus, the H-GNRI may be useful for prognostication in patients with HF. However, further research is necessary to validate its utility in predicting long-term prognosis and assess interventions’ effectiveness for high-risk patients with HF, as indicated by the H-GNRI score.

## Figures and Tables

**Table 1:** Patients’ characteristics.

Characteristics	Overall	Low-Risk Group (H-GNRI Score 2)	Intermediate-Risk Group (H-GNRI Score 1)	High-Risk Group (H-GNRI Score 0)	*p*-Value
Age, years, n [%]					<0.001
- 65–74	460 [13.0]	83 [34.0]	153 [16.1]	224 [9.6]	
- 75–89	2064 [58.4]	139 [57.0]	580 [60.9]	1345 [57.6]	
- ≥90	1008 [28.5]	22 [9.0]	219 [23.0]	767 [32.8]	
Female sex, n [%]	1792 [50.7]	65 [26.6]	440 [46.2]	1235 [52.9]	<0.001
Body mass index, n [%]					<0.001
- <18.5	722 [20.4]	7 [2.9]	72 [7.6]	643 [27.5]	
- 18.5–25	2158 [61.1]	154 [63.1]	640 [67.2]	1364 [58.4]	
- 25–30	605 [17.1]	80 [32.8]	224 [23.5]	301 [12.9]	
- ≥30	47 [1.3]	3 [1.2]	16 [1.7]	28 [1.2]	
New York Heart Association class, n [%]					0.937
- 1	173 [4.9]	10 [4.1]	43 [4.5]	150 [5.1]	
- 2	689 [19.5]	42 [17.2]	188 [19.7]	459 [19.6]	
- 3	1404 [39.8]	103 [42.2]	378 [39.7]	923 [39.5]	
- 4	1129 [32.0]	82 [33.6]	306 [32.1]	741 [31.7]	
- Unclear	137 [3.9]	7 [2.9]	37 [3.9]	93 [4.0]	
Admission to hospital by ambulance, n [%]	1406 [39.8]	102 [41.8]	378 [39.7]	926 [39.6]	0.804
Ventilator at admission, n [%]	452 [12.8]	41 [16.8]	148 [15.5]	263 [11.3]	0.001
Vasopressor at admission, n [%]	542 [15.3]	44 [18.0]	156 [16.4]	342 [14.6]	0.218
Years of admission, n [%]					0.062
- 2017	136 [3.9]	7 [2.9]	37 [3.9]	92 [3.9]	
- 2018	179 [5.1]	5 [2.0]	48 [5.0]	126 [5.4]	
- 2019	266 [7.5]	17 [7.0]	71 [7.5]	178 [7.6]	
- 2020	468 [13.3]	38 [15.6]	131 [13.8]	299 [12.8]	
- 2021	469 [13.3]	42 [17.2]	146 [15.3]	281 [12.0]	
- 2022	2014 [57.0]	135 [55.3]	519 [54.5]	1360 [58.2]	
Number of beds, n [%]					0.006
- 20–99	27 [0.8]	3 [1.2]	9 [0.9]	15 [0.6]	
- 100–199	708 [20.0]	29 [11.9]	189 [19.9]	490 [21.0]	
- 200–299	442 [12.5]	43 [17.6]	131 [13.8]	268 [11.5]	
- 300–499	1179 [33.4]	76 [31.1]	310 [32.6]	793 [33.9]	
- ≥500	1176 [33.3]	93 [38.1]	313 [32.9]	770 [33.0]	
Barthel Index at admission, mean ± SD	50.7 ± 40.29	67.6 ± 39.52	57.1 ± 39.81	46.2 ± 39.76	<0.001
Charlson Comorbidity Index, mean ± SD	2.4 ± 1.48	2.1 ± 1.23	2.3 ± 1.37	2.5 ± 1.54	<0.001

H-GNRI, Hemoglobin-Geriatric Nutritional Risk Index.

**Table 2:** Comparison of outcomes among the three groups.

Outcome Measures	Overall	Low-Risk Group (H-GNRI Score 2)	Intermediate-Risk Group (H-GNRI Score 1)	High-Risk Group (H-GNRI Score 0)	*p*-Value
Barthel Index at discharge, mean ± SD	63.7 ± 40.14	86.2 ± 28.55	72.8 ± 36.26	57.6 ± 41.14	<0.001
Barthel Index gain, mean ± SD	13.1 ± 36.90	18.6 ± 40.39	15.7 ± 36.23	11.4 ± 36.69	<0.001
Barthel Index efficiency, mean ± SD	1.0 ± 4.95	1.7 ± 4.34	1.2 ± 5.34	0.9 ± 4.83	<0.001
Length of hospital stay, mean ± SD	21.5 ± 22.3	15.4 ± 13.7	18.9 ± 19.0	23.3 ± 24.0	<0.001
In-hospital mortality, n [%]	409 [11.6]	10 [4.1]	67 [7.0]	332 [14.2]	<0.001
Discharge to home or nursing home, n [%]	2831 [80.2]	224 [91.8]	814 [85.5]	1793 [76.8]	<0.001
Hospitalization-associated disability, n [%]	541 [15.3]	23 [9.4]	112 [11.8]	406 [17.4]	<0.001

H-GNRI, Hemoglobin-Geriatric Nutritional Risk Index.

**Table 3:** Association between H-GNRI and multiple linear regression analysis outcomes.

Variables	Coefficient	95% Confidence Interval		*p*-Value
		Lower	Upper	
Barthel Index at Discharge				
Low-risk group (reference)	―	―	―	
Intermediate-risk group	−3.87	−8.27	0.51	0.084
High-risk group	−11.08	−15.29	−6.87	<0.001
Barthel Index gain				
Low-risk group (reference)	―	―	―	
Intermediate-risk group	−3.87	−8.27	0.51	0.084
High-risk group	−11.08	−15.29	−6.87	<0.001
Barthel Index efficiency				
Low-risk group (reference)	―	―	―	
Intermediate-risk group	−0.60	−1.27	0.05	0.073
High-risk group	−1.23	−1.87	−0.59	<0.001
Length of hospital stay				
Low-risk group (reference)	―	―	―	
Intermediate-risk group	1.96	−1.12	5.06	0.213
High-risk group	5.29	2.22	8.25	<0.001

Models adjusted for age, sex, New York Heart Association class, Barthel Index at admission, Charlson Comorbidity Index, ventilator use at admission, vasodilator use at admission, use of ambulance service, number of beds, and year of admission.

**Table 4:** Association between H-GNRI and multiple logistic regression analysis outcomes.

Variables	Odds Ratio	95% Confidence Interval		*p*-Value
		Lower	Upper	
In-hospital mortality				
Low-risk group (reference)	―	―	―	
Intermediate-risk group	1.37	0.68	2.77	0.372
High-risk group	2.51	1.28	4.90	0.007
Discharge to home or nursing home				
Low-risk group (reference)	―	―	―	
Intermediate-risk group	0.64	0.38	1.07	0.091
High-risk group	0.42	0.25	0.69	0.001
Hospitalization-associated disability				
Low-risk group (reference)	―	―	―	
Intermediate-risk group	1.13	0.70	1.84	0.602
High-risk group	1.87	1.18	2.97	0.007

Models adjusted for age, sex, New York Heart Association class, Barthel Index at admission, Charlson Comorbidity Index, ventilator use at admission, vasodilator use at admission, use of ambulance service, number of beds, and year of admission.

**Table 5:** Multiple logistic regression analysis for Barthel Index at discharge based on age.

Variables	Coefficient	95% Confidence Interval		*p*-Value
		Lower	Upper	
Age 65–74				
Low-risk group (reference)	―	―	―	
Intermediate-risk group	−2.38	−9.63	4.87	0.519
High-risk group	−4.59	−11.59	2.41	0.198
Age 75–89				
Low-risk group (reference)	―	―	―	
Intermediate-risk group	−5.66	−11.64	0.30	0.063
High-risk group	−14.11	−19.82	−8.41	< 0.01
Age ≥90				
Low-risk group (reference)	―	―	―	
Intermediate-risk group	−9.02	−21.58	3.53	0.159
High-risk group	−15.28	−27.46	−3.11	0.014

Models adjusted for sex, New York Heart Association class, Barthel Index at admission, Charlson Comorbidity Index, ventilator use at admission, vasodilator use at admission, use of ambulance service, number of beds, and year of admission.

**Table 6:** Previous studies of GNRI in patients with heart failure.

Patients	Sample Size	Exclusion Criteria	GNRI Cut off	Outcomes	Conclusions	References
HFpEF	152	･Patients with cancer ･Patients with liver cirrhosis ･Patients on dialysis ･Patients with missing data	92	･all-cause mortality ･HF re-hospitalization ･ADL at discharge	GNRI may be a useful index for predicting functional dependency and mortality	Kinugasa et al. [28]
Acute HF	490	･Patients with acute coronary syndrome ･Patients aged <65 ･Patients with missing for GNRI data	92	･all-cause death ･cardiovascular death ･non-cardiovascular death	GNRI is helpful for risk stratification	Honda et al. [11]
At risk of HF	1823	･Patients without HF risk ･Patients on dialysis	107.1	･cardiovascular events	GNRI may be useful for predicting cardiovascular events	Minamisawa et al. [29]
HFpEF	110	･Patients aged <65 years ･Patients transferred elsewhere ･Patients who died in hospital ･Patients on dialysis ･Patients with missing GNRI data ･Patients other than HFpEF	92	･all-cause mortality	GNRI at discharge is helpful in predicting the long-term prognosis	Nishi et al. [30]
HFpEF	1677	･Patients with missing GNRI data	98	･cardiovascular events ･all-cause death ･HF hospitalization	GNRI was associated with an increased risk for cardiovascular events	Minamisawa et al. [31]
HF	213	･Patients aged <80 years	92	･all-cause death	GNRI could predict poor prognosis in HF hospitalized patients aged ≧80 years	Nakamura et al. [32]
HFpEF/ HFrEF	451	･Patients with acute coronary syndrome ･Patients with active malignancy ･Patients on dialysis ･Patients undergoing surgery during hospitalization ･Patients with missing data	92	･in-hospital mortality ･length of hospital stay	GNRI was not associated with increased in-hospital mortality, GNRI is useful for stratifying patients at high risk for longer length of hospital stay in HFpEF but not in HFrEF	Hirose et al. [9]
HF	91	･Patients with cognitive impairment ･Patients with exercise restrictions ･Patients with a pre-admission Barthel Index of less than 85 ･Patients with missing data	92	･ADL decline	GNRI was not associated with ADL	Kojima et al. [10]

GNRI, Geriatric Nutritional Risk Index; HF, heart failure; HFpEF, heart failure with preserved ejection; ADL, activities of daily life.

## Data Availability

The datasets generated and/or analyzed in this study are available from the corresponding authors upon request.

## References

[1] GBD (2018). Global, regional, and national incidence, prevalence, and years lived with disability for 354 diseases and injuries for 195 countries and territories, 1990–2017: A systematic analysis for the Global Burden of Disease Study 2017. Lancet.

[2] Savarese G., Becher P.M., Lund L.H., Seferovic P., Rosano G.M.C., Coats A.J.S. (2023). Global burden of heart failure: A comprehensive and updated review of epidemiology. Cardiovasc. Res..

[3] Heidenreich P.A., Albert N.M., Allen L.A., Bluemke D.A., Butler J., Fonarow G.C., Ikonomidis J.S., Khavjou O., Konstam M.A., Maddox T.M. (2013). Forecasting the impact of heart failure in the United States: A policy statement from the American Heart Association. Circ. Heart Fail..

[4] Nemoto S., Kasahara Y., Izawa K.P., Watanabe S., Yoshizawa K., Takeichi N., Akao K., Watanabe S., Mizukoshi K., Suzuki N. (2023). Hospital-acquired disability in older heart failure patients decreases independence and increases difficulties in activities of daily living. Eur. J. Cardiovasc. Nurs..

[5] Uemura Y., Shibata R., Takemoto K., Koyasu M., Ishikawa S., Murohara T., Watarai M. (2018). Prognostic impact of the preservation of Activities of Daily Living on post-discharge outcomes in patients with acute heart failure. Circ. J..

[6] Ogawa M., Yoshida N., Nakai M., Kanaoka K., Sumita Y., Kanejima Y., Emoto T., Saito Y., Yamamoto H., Sakai Y. (2022). Hospital-associated disability and hospitalization costs for acute heart failure stratified by body mass index- insight from the JROAD/JROAD-DPC database. Int. J. Cardiol..

[7] Liang L., Zhao X., Huang L., Tian P., Huang B., Feng J., Zhou P., Wang J., Zhang J., Zhang Y. (2023). Prevalence and prognostic importance of malnutrition, as assessed by four different scoring systems, in elder patients with heart failure. Nutr. Metab. Cardiovasc. Dis..

[8] Dong C.H., Chen S.Y., Zeng H.L., Yang B., Pan J. (2021). Geriatric nutritional risk index predicts all-cause mortality in patients with heart failure: A systematic review and meta-analysis. Clinics.

[9] Hirose S., Miyazaki S., Yatsu S., Sato A., Ishiwata S., Matsumoto H., Shitara J., Murata A., Kato T., Suda S. (2020). Impact of the geriatric nutritional risk index on in-hospital mortality and length of hospitalization in patients with acute decompensated heart failure with preserved or reduced ejection fraction. J. Clin. Med..

[10] Kojima I., Tanaka S., Otobe Y., Suzuki M., Koyama S., Kimura Y., Ishiyama D., Maetani Y., Kusumi H., Terao Y. (2022). What is the optimal nutritional assessment tool for predicting decline in the activity of daily living among older patients with heart failure?. Heart Vessels.

[11] Honda Y., Nagai T., Iwakami N., Sugano Y., Honda S., Okada A., Asaumi Y., Aiba T., Noguchi T., Kusano K. (2016). Usefulness of Geriatric Nutritional Risk Index for Assessing Nutritional Status and Its Prognostic Impact in Patients Aged ≥65 Years with Acute Heart Failure. Am. J. Cardiol..

[12] Wang B., Xu C., Ying K., Chu J., Geng W. (2022). Prognostic value of hemoglobin combined with Geriatric Nutritional Risk Index scores in patients undergoing postoperative radiotherapy for esophageal squamous cell carcinoma. Future Oncol..

[13] Anand I.S., Gupta P. (2018). Anemia and Iron Deficiency in Heart Failure: Current Concepts and Emerging Therapies. Circulation.

[14] Martínez-Ruiz A., Tornel-Osorio P.L., Sánchez-Más J., Pérez-Fornieles J., Vílchez J.A., Martínez-Hernández P., Pascual-Figal D.A. (2012). Soluble TNFα receptor type I and hepcidin as determinants of development of anemia in the long-term follow-up of heart failure patients. Clin. Biochem..

[15] Alexandrakis M.G., Tsirakis G. (2012). Anemia in heart failure patients. Int. Sch. Res. Not. Hematol..

[16] Anand I., McMurray J.J., Whitmore J., Warren M., Pham A., McCamish M.A., Burton P.B. (2004). Anemia and its relationship to clinical outcome in heart failure. Circulation.

[17] Polat N., Yıldız A., Bilik M.Z., Aydın M., Acet H., Kaya H., Demir M., Işık M.A., Alan S., Toprak N. (2015). The importance of hematologic indices in the risk stratification of patients with acute decompensated systolic heart failure. Turk. Kardiyol. Dern. Ars..

[18] Nagai K., Tanaka T., Kodaira N., Kimura S., Takahashi Y., Nakayama T. (2020). Data resource profile: JMDC claims databases sourced from Medical Institutions. J. Gen. Fam. Med..

[19] Yasunaga H., Ide H., Imamura T., Ohe K. (2005). Impact of the Japanese Diagnosis Procedure Combination-based Payment System on cardiovascular medicine-related costs. Int. Heart J..

[20] Yamana H., Moriwaki M., Horiguchi H., Kodan M., Fushimi K., Yasunaga H. (2017). Validity of diagnoses, procedures, and laboratory data in Japanese administrative data. J. Epidemiol..

[21] Ouchi Y., Rakugi H., Arai H., Akishita M., Ito H., Toba K., Kai I., Joint Committee of Japan Gerontological Society (JGLS), Japan Geriatrics Society (JGS) on the definition and classification of the elderly (2017). Redefining the elderly as aged 75 years and older: Proposal from the Joint Committee of Japan Gerontological Society and the Japan Geriatrics Society. Geriatr. Gerontol. Int..

[22] Kopelman P.G. (2000). Obesity as a medical problem. Nature.

[23] Mahoney F.I., Barthel D.W. (1965). Functional evaluation: The Barthel index: A simple index of independence useful in scoring improvement in the rehabilitation of the chronically ill. Md. State Med. J..

[24] Charlson M.E., Pompei P., Ales K.L., MacKenzie C.R. (1987). A new method of classifying prognostic comorbidity in longitudinal studies: Development and validation. J. Chronic Dis..

[25] Yamada K., Furuya R., Takita T., Maruyama Y., Yamaguchi Y., Ohkawa S., Kumagai H. (2008). Simplified nutritional screening tools for patients on maintenance hemodialysis. Am. J. Clin. Nutr..

[26] Tsutsumi H., Ohta M. (2006). Diagnosis and treatment of anemia. 4. Anemia of the aged. Nihon Naika Gakkai Zasshi.

[27] Ohta M. (2011). Management of anemia in the elderly. Nihon Naika Gakkai Zasshi..

[28] Kinugasa Y., Kato M., Sugihara S., Hirai M., Yamada K., Yanagihara K., Yamamoto K. (2013). Geriatric nutritional risk index predicts functional dependency and mortality in patients with heart failure with preserved ejection fraction. Circ. J..

[29] Minamisawa M., Miura T., Motoki H., Ueki Y., Nishimura H., Shimizu K., Shoin W., Harada M., Mochidome T., Senda K. (2018). Geriatric Nutritional Risk Index Predicts Cardiovascular Events in Patients at Risk for Heart Failure. Circ. J..

[30] Nishi I., Seo Y., Hamada-Harimura Y., Yamamoto M., Ishizu T., Sugano A., Sato K., Sai S., Obara K., Suzuki S. (2019). Geriatric nutritional risk index predicts all-cause deaths in heart failure with preserved ejection fraction. ESC Heart Fail..

[31] Minamisawa M., Seidelmann S.B., Claggett B., Hegde S.M., Shah A.M., Desai A.S., Lewis E.F., Shah S.J., Sweitzer N.K., Fang J.C. (2019). Impact of Malnutrition Using Geriatric Nutritional Risk Index in Heart Failure With Preserved Ejection Fraction.. JACC Heart Fail..

[32] Nakamura T., Matsumoto M., Haraguchi Y., Ishida T., Momomura S.I. (2020). Prognostic impact of malnutrition assessed using geriatric nutritional risk index in patients aged ≥80 years with heart failure. Eur. J. Cardiovasc. Nurs..

[33] Hsieh Y.W., Wang C.H., Wu S.C., Chen P.C., Sheu C.F., Hsieh C.L. (2007). Establishing the minimal clinically important difference of the Barthel Index in stroke patients. Neurorehabilit. Neural Repair..

[34] Unnanuntana A., Jarusriwanna A., Nepal S. (2018). Validity and responsiveness of Barthel index for measuring functional recovery after hemiarthroplasty for femoral neck fracture. Arch. Orthop. Trauma Surg..

[35] Maraldi C., Volpato S., Cesari M., Cavalieri M., Onder G., Mangani I., Woodman R.C., Fellin R., Pahor M. (2006). Anemia and recovery from disability in activities of daily living in hospitalized older persons. J. Am. Geriatr. Soc..

[36] Saitoh M., Ozawa T., Okamura D., Mabuchi M., Nakazawa M., Aoyagi M., Sakamoto J., Akiho M. (2014). Effects of let ventricular systolic dysfunction and cardio-renal anemia syndrome on walking ability and Activities of Daily Living in patients with heart failure. Phys. Ther. Jpn..

[37] Saitoh M., Itoh H., Morotomi N., Ozawa T., Ishii N., Uewaki R., Hori K., Shiotani Y., Ando M., Nakashima S. (2014). Impact of chronic kidney disease and anemia on physical function in patients with chronic heart failure. Cardiorenal Med..

[38] Listerman J., Geisberg C., Nading M.A., Goring J., Huang R., Butler J. (2007). Blunted hemodynamic response and reduced oxygen delivery with exercise in anemic heart failure patients with systolic dysfunction. Congest. Heart Fail..

[39] Hirani V., Naganathan V., Blyth F., Le Couteur D.G., Seibel M.J., Waite L.M., Handelsman D.J., Hsu B., Cumming R.G. (2016). Low hemoglobin concentrations are associated with sarcopenia, physical performance, and disability in older Australian men in cross-sectional and longitudinal analysis: The concord health and ageing in men project. J. Gerontol. A Biol. Sci. Med. Sci..

[40] Wakabayashi H., Sashika H. (2014). Malnutrition is associated with poor rehabilitation outcome in elderly inpatients with hospital-associated deconditioning a prospective cohort study. J. Rehabil. Med..

[41] Wakabayashi H., Sakuma K. (2014). Rehabilitation nutrition for sarcopenia with disability: A combination of both rehabilitation and nutrition care management. J. Cachexia Sarcopenia Muscle.

[42] Huang C.H., Hsu C.C., Yu P.C., Peng L.N., Lin M.H., Chen L.K. (2021). Hospitalization-associated muscle weakness and functional outcomes among oldest old patients: A hospital-based cohort study. Exp. Gerontol..

[43] de Luis D.A., Culebras J.M., Aller R., Eiros-Bouza J.M. (2014). Surgical infection and malnutrition. Nutr. Hosp..

[44] Gladysheva I.P., Sullivan R.D., Pellicori P. (2023). Editorial: Edema in heart failure with reduced ejection fraction. Front. Cardiovasc. Med..

[45] Lee K.S., Kim J.H., Kang J., Cho H.J., Lee H.Y. (2023). Association between Changes in Bioelectrical Impedance Analysis (BIA) Parameter and the Clinical Outcomes in Patients with Acute Heart Failure. J. Korean Med. Sci..

[46] Saito H., Matsue Y., Maeda D., Kasai T., Kagiyama N., Endo Y., Zoda M., Mizukami A., Yoshioka K., Hashimoto T. (2022). Prognostic values of muscle mass assessed by dual-energy X-ray absorptiometry and bioelectrical impedance analysis in older patients with heart failure. Geriatr. Gerontol. Int..

